# Identifying Patterns in Dental Visit Attendance Among Pregnant Women: A Retrospective Cohort Study

**DOI:** 10.1016/j.focus.2025.100322

**Published:** 2025-02-07

**Authors:** Nisreen Al Jallad, Samantha Manning, Xinyue Mao, Parshad Mehta, TongTong Wu, Rita Cacciato, Jiebo Luo, Yihong Li, Jin Xiao

**Affiliations:** 1 Eastman Institute for Oral Health, University of Rochester Medical Center, Rochester, New York; 2 Department of Biostatistics and Computational Biology, University of Rochester Medical Center, Rochester, New York; 3 Master of Public Health Program, Department of Public & Ecosystem Health, Cornell University, Ithaca, New York; 4 Department of Computer Science, University of Rochester Medical Center, Rochester, New York

**Keywords:** Pregnancy, prenatal oral healthcare, social determinants of health, care-seeking patterns

## Abstract

**Introduction:**

Understanding the factors influencing dental care utilization is crucial for enhancing treatment adherence and outcomes. This study evaluates dental care–seeking patterns among pregnant women in low-income community.

**Methods:**

The authors analyzed data from 311 pregnant patients and 1,111 visits (2019–2022) synchronized from dental and medical records. The primary outcome was showing up for scheduled dental visits. To identify visit-attending patterns, the authors used a model-based clustering method to cluster longitudinal data with categorical outcomes. A penalized generalized linear mixed-effects model was applied to identify relevant variables for the visit attendance trajectories within each cluster.

**Results:**

The study participants comprised 49.6% Black, 32.2% White, and 12.5% Hispanic women. The majority (89.07%) were holding Medicaid insurance. Among the 1,111 scheduled visits, 432 resulted in no-shows (38.8%), including failed and canceled appointments. The authors identified 3 distinct clusters of visit-attending patterns on the basis of their show-up rates: low demand/low appointment risk (85% attendance), high demand/high appointment risk (57% attendance despite multiple scheduled visits), and moderate demand/high appointment risk (55% attendance with fewer scheduled visits). Various determinants, such as race; age; inner-city residence; appointment timing; the COVID-19 era; type of scheduled dental treatment; and prior medical visits for conditions such as anxiety, depression, hypertension, and allergies, influenced the visit-attending behaviors within each patient group.

**Conclusions:**

The innovative clustering approach of this study successfully identified dental care–seeking patterns among pregnant women, suggesting its applicability to a broader demographic. Identifying potential modifiable factors that could enhance attendance at dental visits is essential for improving oral healthcare outcomes among underserved pregnant patients.

## HIGHLIGHTS

• This study examined dental care–seeking patterns in 311 underserved pregnant women.
• Modifiable factors were identified to improve visit attendance in underserved groups.
• Three clusters were identified: low demand/low risk, high demand/high risk, and moderate demand/high risk.
• Determinants were race, age, residence, appointment timing, COVID-19 status, and treatment type.
• Anxiety, depression, hypertension, and allergies influenced care-seeking behaviors.

## INTRODUCTION

Dental care–seeking behaviors vary widely among different populations, reflecting disparities in access to dental services. In the U.S., a significant portion of dental appointments, approximately 23.4%, are canceled, and 6.6% result in failure to attend.^1^ In addition, the coronavirus disease 2019 (COVID-19) pandemic has caused significant delays in patients receiving treatment for dental conditions.^2^ As reported, over 26% of households missed dental appointments for at least 1 child or adolescent owing to the COVID-19 pandemic.^3^

Patient no-shows in the healthcare system, particularly in dental care, have profound implications.^4^ This problem is not merely about financial loss; it extends to broader consequences that affect the healthcare system's efficiency, patient health outcomes, and the operational dynamics of healthcare facilities.^5^ Regular dental visits are critical for maintaining oral health, allowing for the prevention, early detection, and treatment of dental issues. No-shows interrupt this care continuum, increasing the risk of undiagnosed and untreated dental problems. This neglect can escalate into more severe health issues, necessitating more complex and expensive treatments down the line, underscoring the importance of consistent patient attendance.^6^ For dental practices, no-shows create scheduling inefficiencies, leaving gaps that could have been allocated to other patients. This disruption affects the practice's ability to optimize care delivery and maintain operational efficiency, leading to potential revenue losses and decreased ability to serve the community effectively.^7^ The cycle of missed appointments can frustrate both patients and dental practices. Patients may experience delays in receiving necessary care, leading to dissatisfaction with the service and potentially impacting their loyalty to the practice.^8^

Pregnancy represents a unique and complex period in a woman's life, marked by significant physiologic and biological changes that can increase susceptibility to oral diseases such as gingivitis, periodontitis, dental caries, and xerostomia.^9^^,^^10^ Neglecting oral care during pregnancy not only affects the mother's oral health but also has implications for the infant's well-being.^10^ Despite the demonstrated safety of routine oral health care during pregnancy and professional guidelines recommending it,^11^^,^^12^ utilization of prenatal oral healthcare remains low in New York state and the nation at large.^13^^,^^14^ Alarmingly, more than half of pregnant women do not seek dental care during pregnancy despite recognizing its importance.^13^^,^^14^ In the U.S., only 22%–34% of pregnant women visit a dentist.^15^

Understanding the patterns of dental care–seeking behavior among pregnant patients and identifying modifiable factors that could encourage patients’ attendance at dental appointments are crucial for enhancing oral healthcare outcomes in underserved pregnant populations and ultimately reducing oral health disparities.^15^, ^16^, ^17^, ^18^ Developing a predictive model to understand why pregnant women frequently miss dental appointments could provide valuable insights into the obstacles they encounter. This understanding could refine appointment scheduling, better address patient needs, and ultimately mitigate oral health disparities among pregnant women. Therefore, this study aimed to assess dental care–seeking patterns among pregnant women from a low-income and minoritized underserved community in the U.S. and further identify factors associated with prenatal dental care–seeking behaviors.

## METHODS

### Study Population

This retrospective cohort study included patients of University of Rochester Medical Center Perinatal Dental Clinic from January 1, 2019, to December 31, 2022. The University of Rochester Medical Center includes entities that provide medical and dental care. The medical and dental records of eligible participants were used for data collection. The study was approved by the University Research Subject Review Board (STUDY00007883).

The inclusion criteria were (1) pregnant female, (2) being aged ≥18 years, and (3) having both dental and medical electronic records in the system. The exclusions criteria were pregnant women whose oral health care was contraindicated.

### Measures

A total of 311 patients were included in this study. Data were obtained using dental records from the axiUm electronic system and medical records from the EPIC eRecord. The following variables were obtained:

1. Demographic and socioeconomic factors: race (Black, White, and others), ethnicity (Hispanic, non-Hispanic, and unknown/unreported), age (continuous), residency (living in the inner city or not), and insurance type (Medicaid and non-Medicaid);
2. Types of dental appointments (new patient examination, recall examination, oral hygiene, dental treatment, emergency assessment), date and time of dental appointments, and scheduled treatment (emergency, examination, endodontic, prophy and periodontal, restorations, surgery, treatment plan, and undefined visits); and
3. Medical history: hypertension, diabetes (gestational, Type I, and Type II), anxiety, depression, kidney disease, and allergies (medicine, food, and environmental). The severity levels for allergies included low, median, and high.

The medical history data were synchronized into the dental visit data by defining ever variables for all groups of medical conditions. In the merged dataset, with each row meaning 1 scheduled dental appointment, the ever variables answer the questions such as whether the patient had ever had depression before this dental visit. Apart from such yes or no questions, there was interest in the frequency of one patient's medical visit for whatever purposes. Thus, counts of visits during the last week, last month, and last year were generated from medical history. Smoking was also defined as an ever variable, indicating whether the patient had ever smoked before the scheduled dental visit.

The primary outcome was the status of dental visit attendance. Three types of attendance were obtained from the dental records, including successfully checked out, canceled, or failed to show. For this study, the authors defined the outcome as a binary response, with 0 indicating canceled or failed and 1 indicating a successful visit.

### Statistical Analysis

The authors used the model-based clustering method introduced by Manning et al.^19^ developed from the method proposed by Yang and Wu.^20^ This method clusters trajectories in longitudinal data with categorical responses while also reporting significant covariates relevant to the assignment of the clusters. It allows for the identification of latent heterogeneous subpopulations in the data, which can evoke a better understanding of the relationship between the independent data and the response.

In this study, the authors aimed to cluster this sample of pregnant females on the basis of their dental visit attendance patterns over time. There were 3 possible records of attendance: successfully checked out, canceled, or failed to showing. The outcome analysis enabled the authors to identify influential factors that altered dental visit attendance rates. The first step in the analysis was to initialize the clustering assignment with a k-means clustering analysis on the covariates. Then, the model-based trajectory clustering algorithm was implemented with 2 alternating stages.

First, a GLMM-LASSO (generalized linear mixture model with the least absolute shrinkage and selection operator) penalization was fit to each cluster according to the previous clustering step. This accounts for the longitudinal nature of the data with a random intercept for each subject because these observations will be necessarily correlated. The model was fit using the glmmLasso package^20^ in R. Because the response variable was binary, the logit link function was used in the model. To choose the optimal penalty parameter lambda, which determines the number of nonzero variables in the model, the Akaike information criterion was used. The Akaike information criterion of all models in a given range of lambda values was calculated for each iteration to permit greater flexibility, which is necessary because of the fluctuating cluster sizes and compositions across each iteration of the algorithm. To avoid the bias inherent in a least absolute shrinkage and selection operator (LASSO) regression model, the nonzero coefficients (the active set) of the model were re-estimated using an unpenalized generalized linear mixture model, using the glmer function of the lme4 package in R. This final re-estimated model was used to calculate the estimated probabilities of each subject successfully completing their visit at each time point (the fitted values). These fitted values were used to calculate new cluster assignments in the second stage of the algorithm. The other outputs from the first stage are the active set of each cluster's GLMM-LASSO model. These coefficients indicate which variables were important in determining cluster membership.

The second stage of the algorithm determines cluster membership. The estimated probabilities were used as the input of a k-means procedure for longitudinal response trajectories^21^ (KmL), with k being the number of clusters. This accounts for the correlated nature of repeated measures. Because not all subjects had the same number of observations, the Gower adjustment was used. The Gower adjustment, in the Euclidean case as used in this study, takes 2 vectors of different lengths and calculates the distance between them using only coordinates at which both vectors do not have missing values. This avoids issues with unequal amounts of observations per subject. The KmL algorithm clusters estimated the probabilities of similar trajectories together to generate new cluster assignments. These new cluster assignments are used to repeat the first stage of the algorithm. The 2 stages were alternated until cluster membership remained constant. The clustering analysis determined that there were 3 clusters in the sample.

## RESULTS

Among the 311 pregnant patients, 32.2% were White, 49.6% were Black, and 19.2% belonged to other racial groups. In addition, 12.5% of the pregnant women identified as Hispanic. Table 1 presents the characteristics of the study cohort. There was a significant increase in the number of pregnant women seeking dental care after COVID-19 pandemic, nearly doubling the figures for those who sought care before or during the pandemic, as illustrated in Table 1. Furthermore, it is noteworthy that there has been a demographic shift, with pregnant women from racial groups other than Black or White displaying the highest inclination toward initiating dental care after the COVID-19 period.

**Table 1:** Demographic–Socioeconomic–Behavior–Medical–Dental Conditions

Variables	All patients N=311	Before COVID-19 (January 1, 2019–March 15, 2020) *n*=86	During COVID-19 (March 16, 2020–March 15, 2021) *n*=78	After COVID-19 (March 16, 2021–December 31, 2022) *n*=147
Age group, years				
18–30	63.02%	66.28%	64.10%	60.54%
>30	36.98%	33.72%	35.90%	39.45%
Race				
White	32.15%	39.53%	28.21%	29.93%
Black or African American	49.20%	47.67%	55.13%	46.84%
Others	18.65%	12.80%	16.66%	23.23%
Hispanic (Y)	12.54%	9.30%	12.82%	14.29%
Inner City Residence (Y)	76.21%	74.42%	79.48%	75.5%
Monroe county (Y)	89.39%	86.05%	92.31%	89.80%
Medicaid insurance (Y)	89.07%	90.70%	89.74%	87.76%
Diabetes (Y)	7.7%	5.81%	7.69%	9.52%
Anxiety (Y)	43.09%	40.70%	42.31%	45.58%
Depression (Y)	43.73%	38.37%	46.15%	47.62%
High blood pressure (Y)	15.76%	22.09%	11.54%	8.84%
Smoking (Y)	19.29%	18.60%	19.23%	19.73%

*Note:* The column All patients summarizes the baseline information for all subjects. The medical condition variables and Smoking (Y) consider subjects who have answered yes for the corresponding records. The other 3 columns present the same information from different periods. A subject was categorized into before COVID-19/during COVID-19/after COVID-19 if her first visit was before COVID-19, during COVID-19, or after COVID-19.

**Figure 1:**
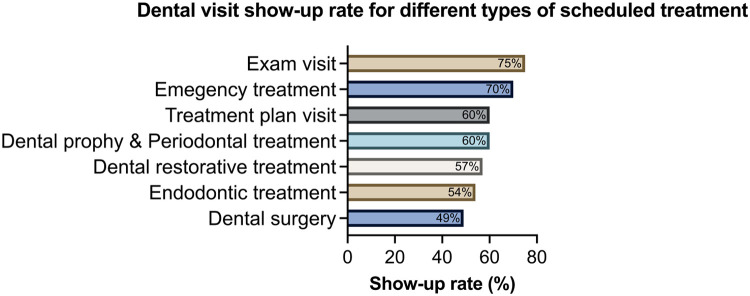
Dental visit show-up rate for different types of scheduled treatment. Dental visits with various purposes have significantly different show-up rates (chi-square test of show-up rate among different types of scheduled treatment, *p*=1.907 × 10^−5^). Dental visits for examination visits had the highest show-up rate (75%), followed by those for emergency treatment (70%). Dental surgery had the lowest show-up rate (49%).

**Figure 2:**
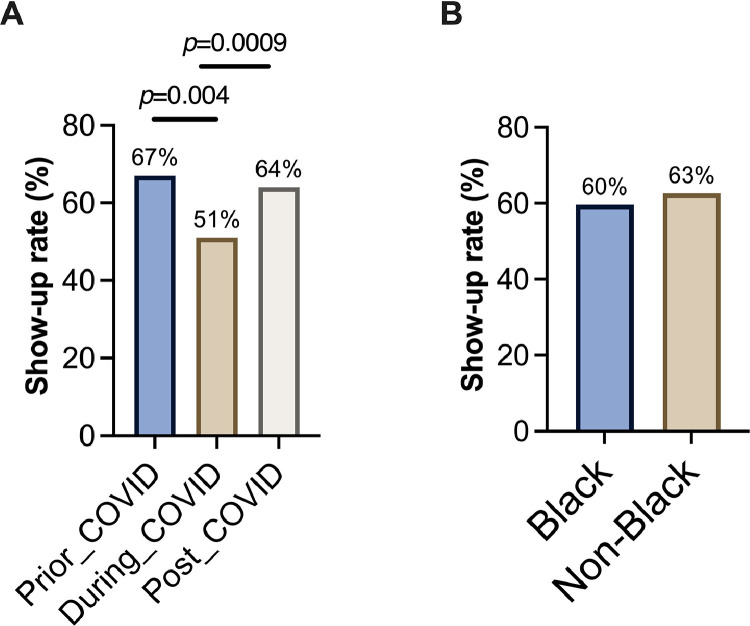
Attendance of dental visits by the COVID-19 pandemic and race. (A) The show-up rates prior to, during, and after COVID-19 were significantly different. There was clearly a trend that the show-up rate decreased from 67% prior to COVID-19 to 51% during the COVID-19 and increased to 64% after COVID-19. Pearson's chi-square test yields *p*=3.02 × 10^−5^, indicating a statistically significant difference among the 3 periods. (B) The show-up rates were similar between Black and non-Black groups.

**Figure 3:**
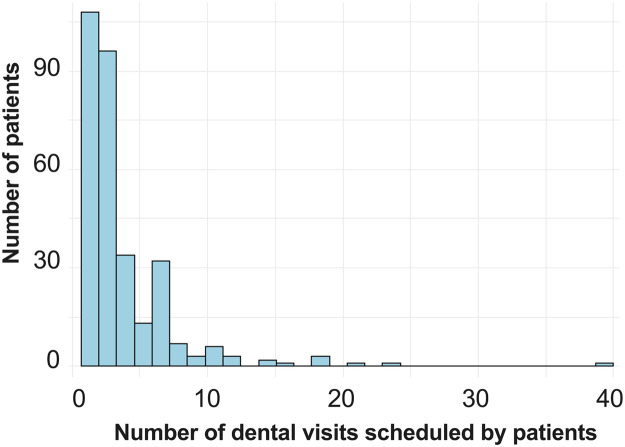
Number of dental visits scheduled by patients. The histogram shows that 108 patients of 311 have only 1 scheduled appointment. There were 96 subjects with 2 or 3 visits.

Of the 1,111 visits involving 311 subjects, 181 failed to show, and 251 canceled visits, resulting in 432 no-shows (38.8%) and 679 attended visits (69.1%). Show-up rates varied significantly across different types of scheduled treatments (*p*<0.00001), as depicted in Figure 1. Dental examinations had the highest show-up rate (75%), followed by emergency treatments (70%). However, appointments scheduled for surgery had a notably lower show-up rate at 49%. Show-up rates differed significantly before, during, and after the COVID-19 pandemic (*p*=3.02 × 10^−5^) (Figure 2A). Show-up rates declined from 67% before COVID-19 to 51% during COVID-19 and then increased to 64% after COVID-19. No racial disparities were observed in show-up rates (Figure 2B); Black and non-Black pregnant women exhibited similar attendance rates (*p*=0.35). The histogram in Figure 3 illustrates that 108 patients of the total 311 had only 1 scheduled appointment, whereas 96 subjects had 2 or 3 visits. In addition, a few patients scheduled >20 visits.

The algorithm resulted in 3 clusters, with the key summary statistics regarding the final cluster assignments provided in Table 2. The 3 clusters had distinct visit-attending patterns: low demand/low appointment risk (85% attendance), high demand/high appointment risk (57% attendance despite multiple scheduled visits), and moderate demand/high appointment risk (55% attendance with fewer scheduled visits). Cluster 1 (low demand/low appointment risk) primarily consisted of participants with the highest show-up rates. In contrast, Clusters 2 (high demand/high appointment risk) and 3 (moderate demand/high appointment risk) had participants who were more likely to fail or cancel their visit. The primary difference between Clusters 2 and 3 regarding their dental visit patterns was that the subjects in Cluster 2 had many more visits scheduled per subject, an average of 13.56 visits per patient. It is worth investigating these clusters further because they indicate separate and relevant dental visit tendencies.

**Table 2:** Clusters Generated by GLMM-LASSO Clustering Method

Visits and subject information	Cluster 1 (low demand/low appointment risk)	Cluster 2 (high demand/high appointment risk)	Cluster 3 (moderate demand/high appointment risk)
Canceled/failed visits	34	145	240
Successful visits	191	194	288
Total visits	225	339	528
Total subjects	166	25	117
Average success rate	85%	57%	55%
Average visits per subject	1.35	13.56	4.5

GLMM-LASSO, generalized linear mixture model with the least absolute shrinkage and selection operator.

The algorithm also generated cluster-specific coefficients, provided in Table 3. These coefficients indicate what factors were important to show up in a dental visit in the corresponding cluster. A negative covariate indicates that the presence of the variable in question indicates lower odds of the subject attending a dental visit. A positive sign indicates the opposite. Several factors, such as the patient's age and residing in the inner city, were identified in multiple clusters, although they had different coefficient values in each. There were also variables unique to each cluster. For example, environmental allergy, selected in Cluster 3, contributed negatively to the odds of a successful visit.

**Table 3:** Estimated Coefficients From Cluster-Specific GLMM-LASSO Models

Variables	Coefficients
Cluster 1 (low demand/low appointment risk)	
Black	−0.228
White	−0.187
Inner city	−0.009
Age	0.060
After COVID-19	0.416
Mid-morning visit	0.432
Early afternoon visit	0.662
Restoration visit	−0.371
Depression before visit	−0.434
Anxiety before visit	0.135
Visit last year	−0.010
Medical severity	0.260
Winter appointment	0.461
Cluster 2 (high demand/high appointment risk)	
Inner city	0.182
Age	0.003
Early-morning visit	0.411
Late-morning visit	0.745
Restoration visit	0.104
Anxiety before visit	0.065
Diabetes II before visit	0.357
Winter appointment	0.072
Spring appointment	−1.217
Summer appointment	0.091
Cluster 3 (moderate demand/high appointment risk)	
Inner city	0.033
Age	0.034
After COVID-19	−0.115
During COVID-19	−0.564
Restoration visit	−0.559
Surgery visit	−0.714
Depression before visit	−0.034
Anxiety before visit	−0.292
Hypertension before visit	−0.289
Visit last year	−0.004
Environmental allergy	−0.245
Spring appointment	−0.328
Summer appointment	0.307

GLMM-LASSO, generalized linear mixture model with the least absolute shrinkage and selection operator.

**Figure 4:**
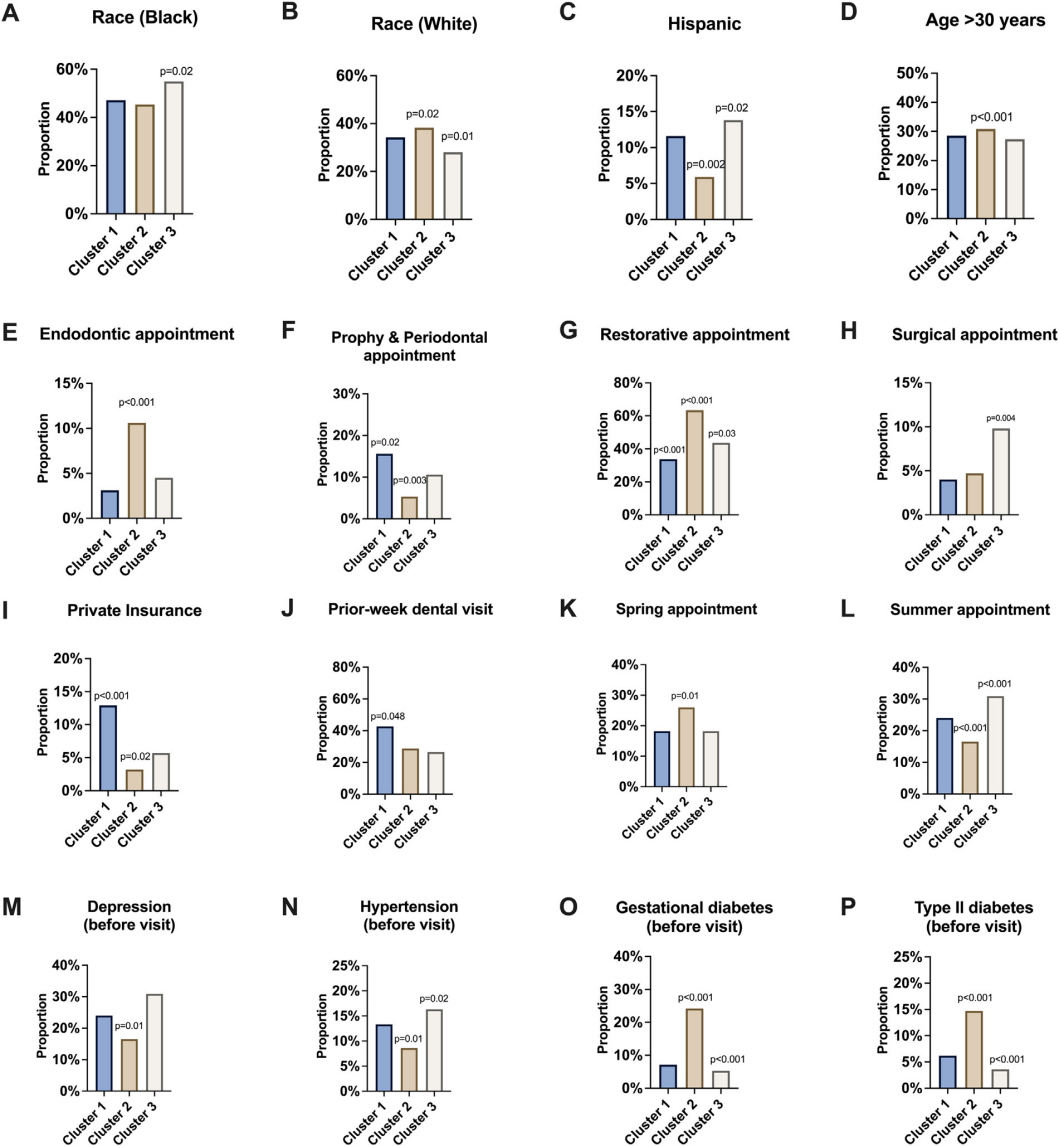
Significantly different variables were identified between the 3 clusters. Postclustering analysis revealed variables that exhibited significant differences between clusters. The listed *p*-value above each cluster mean indicates that this cluster significantly differs from the other 2 clusters with respect to this variable.

Furthermore, a postclustering analysis was performed. It took the final cluster assignments and determined the differences between the discovered groups. The authors examined all variables between the 3 clusters to see whether any were significantly different between or across the clusters. To ensure that the false discovery rate was controlled, the Benjamini–Hochberg procedure was performed on all *p*-values of the comparisons. Figure 4 contains bar plots of the significantly different variables across clusters, in addition to an indicator of their *p*-value range after adjustment. The postclustering analysis likely reflects the phase of disease management or treatment for pregnant women: health-maintenance phase (Cluster 1), restorative phase (Cluster 2), and surgical/urgent phase (Cluster 3). For example, the variable of prophy-perio visit, meaning a dental cleaning or periodontal treatment, showed a significantly larger proportion in Cluster 1 and a significantly smaller proportion in Cluster 2. In contrast, those whose scheduled visit was a dental restoration had a significantly higher proportion in Cluster 2. The results demonstrated that the show-up rate for restorations is poor, but further insight would say that those who have a propensity to fail to attend dental visits have poorer oral health, resulting in a greater need for restorative treatments.

## DISCUSSION

Addressing the issue of no-shows requires a multifaceted approach, including better patient education on the importance of dental health, implementing reminder systems, and possibly considering policies that mitigate the impact of missed appointments. Improving access to care and patient–practice communication can also play a significant role in reducing no-show rates and enhancing the overall effectiveness of healthcare delivery. Some studies have explored the determinants influencing dental care–seeking behaviors among pregnant women, primarily focusing on common factors such as SES and insurance coverage. However, to the best of the authors' knowledge, this study represents the first to establish a connection between patients’ medical visitation patterns, driven by their health status and their attendance at dental clinics.

Furthermore, currently, no method offers simultaneous clustering and variable selection of longitudinal, high-dimensional data with a categorical outcome. The method finds clusters using the information deemed relevant by the GLMM-LASSO penalized model, so the connection between the clusters and the selected variables is unambiguous. Other methods that handle high-dimensional data will often filter out potentially irrelevant variables first and then perform clustering, which ignores the fact that different clusters will consider different variables to be of relevance. The modeling developed in this study is relatively superior to performing clustering and only fitting a model because the authors used only the most relevant information to determine clusters, making the clustering itself much more reliable. Moreover, the LASSO component in GLMM-LASSO enhances model performance by reducing overfitting and improving interpretability, particularly in high-dimensional datasets with many predictors, where traditional models, such as multinomial logistic regression, may struggle owing to overfitting or computational challenges.

The authors observed a significant increase in the number of pregnant women seeking dental care after the COVID-19 pandemic, nearly doubling the number for those who had their first appointment before or during the pandemic (Table 1). This trend may reflect a backlog in dental care needs due to many dental clinics in the U.S. being closed or operating at reduced capacity during the early stages of COVID-19. In addition, there was a demographic shift in the authors’ clinic's post–COVID-19 pregnant patient population, with an increase in patients from non-Black and non-White racial groups. The authors also noted a lower proportion of pregnant women with hypertension in the postpandemic cohort, suggesting that those seeking care at the perinatal dental clinic after COVID-19 may be, on average, healthier than those seen before or during the pandemic.

For patients with longitudinal data already collected, the authors can assign cluster membership by fitting their data to each cluster-specific models and choosing the one that generates the highest estimated probability. To assign cluster membership to a patient who only has baseline data, the authors first fit a multinomial model to the baseline data of the sample, using only variables that were selected to be significantly different between clusters and could reasonably be assumed to have been collected as baseline information. Then, the authors assigned the subject to the cluster with the highest probability, say, for example, a White woman aged 22 years who lives in the inner city and has Type II diabetes and experienced diabetes in pregnancy. With this model, the probabilities of her being in Clusters 1 (low demand/low appointment risk), 2 (high demand/high appointment risk), and 3 (moderate demand/high appointment risk) would be 31%, 56%, and 13%, respectively, so the authors would assign her to Cluster 2 (high demand/high appointment risk). From there, using only the baseline data in the Cluster 2–specific model, the authors estimate that her probability of attending her appointment is 64.5%. To increase this probability and enhance appointment attendance, it would be advised to schedule the dental appointment either in the winter or summertime as well as either in the early or late morning, as indicated by the positive coefficients in the cluster-specific model for Cluster 2. The authors acknowledge that the implications for improving attendance in dental care are based on data from the current preliminary model. This model will be refined as more data become available, offering additional modifiable measures to enhance care.

In addition, the authors observed that patients in Clusters 2 and 3 appeared to have more complex medical profiles than those in Cluster 1. Patients in Cluster 2 had a higher frequency of diabetes-related visits before their dental appointments, whereas those in Cluster 3 had more visits related to depression and hypertension. Both Clusters 2 and 3 showed a higher likelihood of missed dental appointments, potentially reflecting the Medical Center's role as a regional hub for managing the oral health needs of low-income patients with complex medical histories.

### Limitations

The following limitations should be considered when interpreting the study results. The study results might not be generalized to other populations outside Upstate New York. Patients who visit the authors’ medical facility comprise approximately 40% Black, 40% White, and 20% others. The dataset included the period across the COVID-19 pandemic; care-seeking behavior among patients will be impacted by national and regional healthcare guidelines during the COVID-19 pandemic, such as social distancing, not attending medical visits when with suspicious COVID-19 symptoms. The data collected on medical conditions were measured as binary variables. Hence, the magnitude and severity of those conditions were not considered while estimating the potential confounding effects. In addition, this dataset lacked information on family structure, such as household size, marital status of pregnant women, and pregnancy trimester. Future studies incorporating these social determinants of health and detailed pregnancy information could offer a more targeted approach to addressing barriers to prenatal dental visits among underserved pregnant women. Finally, the clustering method is a novel unsupervised method, which in general requires further investigation to refine its biological interpretability. However, it may have limitations in detecting variables with low within-cluster variation, which could impact the comprehensiveness of the analysis.

Future studies are needed to further develop the preliminary models presented in this paper. This approach holds potential for enhancing population-based healthcare management by generating hypotheses and testing interventions aimed at supporting pregnant women in need of dental care.

## CONCLUSIONS

Understanding dental care–seeking patterns among pregnant patients in underserved communities and potential modifiable factors that could facilitate attendance at dental visits are the keys to improving oral healthcare accessibility, utilization, and outcomes, ultimately reducing oral health disparities in these populations**.**
